# Navigation delivery models and roles of navigators in primary care: a scoping literature review

**DOI:** 10.1186/s12913-018-2889-0

**Published:** 2018-02-08

**Authors:** Nancy Carter, Ruta K. Valaitis, Annie Lam, Janice Feather, Jennifer Nicholl, Laura Cleghorn

**Affiliations:** 1 McMaster University, School of Nursing, Hamilton, Canada; 2 University of Toronto, Faculty of Nursing, Toronto, Canada

**Keywords:** System navigation, Patient navigation, Navigator, Primary health care, Scoping literature review, Models of care, Community health, Social services

## Abstract

**Background:**

Systems navigation provided by individuals or teams is emerging as a strategy to reduce barriers to care. Complex clients with health and social support needs in primary care experience fragmentation and gaps in service delivery. There is great diversity in the design of navigation and a lack of consensus on navigation roles and models in primary care.

**Methods:**

We conducted a scoping literature review following established methods to explore the existing evidence on system navigation in primary care. To be included, studies had to be published in English between 1990 and 2013, and include a navigator or navigation process in a primary care setting that involves the community- based social services beyond the health care system.

**Results:**

We included 34 papers in our review, most of which were descriptive papers, and the majority originated in the US. Most of the studies involved studies of individual navigators (lay person or nurse) and were developed to meet the needs of specific patient populations. We make an important contribution to the literature by highlighting navigation models that address both health and social service navigation. The emergence and development of system navigation signals an important shift in the recognition that health care and social care are inextricably linked especially to address the social determinants of health.

**Conclusions:**

There is a high degree of variance in the literature, but descriptive studies can inform further innovation and development of navigation interventions in primary care.

## Background

The need for patient navigation is in response to the growing complexity of healthcare service delivery, the aging population, increased polymorbidity, and social inequalities in population health. Patient navigation programs are far from stagnant – dynamic changes to healthcare service delivery have contributed to the emergence of patient navigation in many facets of the healthcare system. Patient navigation programs emerged in the United States and Canada in the 1990s, initially to increase cancer screening rates in underserved populations [1, 2]. Since then, system navigation is regarded by many as a key component of supportive cancer care for all populations [1, 3–5]. Patient navigation is intended as a method of barrier reduction, bridging gaps in service which serve as pitfalls for complex patients. The central point of access in the system for complex patients should be primary care [6]. Medically complex patients in primary care experience fragmentation and gaps in service delivery, primary care reforms involving system navigation may support the management of this patient population [7–9]. Navigators assist with fragmentation of the health and social health care system through various methods including: communication with multiple agencies [10–12], facilitating access to care [10, 13, 14], navigating the system and services [13, 15, 16], or assisting individuals with health insurance. Primary care lends itself well to the concept of system navigation, since one of its key features is the coordination of care across health care providers and services in the interest of person-centred care [17, 18]. In spite of this moderate groundswell of support for system navigation, the merits of these programs are debated in research and practice, as system navigation is often viewed as a “band-aid” solution that diverts attention away from the need for system-level changes to improve care coordination and integration across healthcare systems in the United States and Canada [19–22].

While this debate remains unsolved, system navigation programs continue to be developed and evaluated in North America and internationally. Due to the lack of consensus in program development it is not surprising that the literature reveals great diversity in the design and target populations for these programs [e.g., race, gender, age, socioeconomic status, geographic location or disease group (cancer, HIV, mental illness, addictions, “complex”)], which is also reflective of the great diversity of primary care models and professional roles. There is also significant variability in the parameters of the navigator role, scope of practice and models of care, as navigators can be trained lay persons or healthcare professionals from a variety of disciplines, and they can work as individuals or as part of a team.

This scoping literature review explores the existing evidence on system navigation in primary care, conceptualizing navigation that is inclusive of the linkages with community-based health and social services (CBHSS). In other words, we specifically explore navigation that extends beyond medical specialists and hospitals to capture the provision of social care. CBHSS are often unused by primary care due to lack of awareness [23]. In the US, 80% of physicians are not confident in providing for social needs of their patients such as access to nutritious food, transportation and housing [24]. At the same time they understand that unmet social needs negatively influence health not only for those in low-income communities. It is well known that the social determinants of health must be addressed to achieve health equity [25].

Results will increase the understanding of characteristics of models and frameworks of primary care system navigation and their impacts, as well as the variability of system navigator roles, and populations served. There is great utility in assessing components and features of effective navigation models and of the role itself, to determine whether these programs are a temporary fix or a necessity to achieve comprehensive care for socially and/or medically complex patients in primary care. A separate publication focuses on the topic of implementing these navigation models in primary care [26].

Given the range of approaches that characterize navigation programs, there is no commonly accepted definition of system or patient navigation. For our review, we consider system navigation to refer to an individual or a team engaging in specific activities that include the following concepts: 1) facilitating access to health-related programs and social services for patients/families and caregivers; 2) promoting and facilitating continuity of care; 3) identifying and removing barriers to care; and 4) effective and efficient use of the health care system for both patients/families, caregivers and practitioners [1, 7, 27, 28]. In the case where an individual occupies a navigation role, this person can be a health care professional, or a non-professional (lay person) who is trained to perform specific activities related to system navigation functions.

Primary care in this review is defined as “a service at the entrance to the healthcare system. It addresses diagnosis, ongoing treatment and the management of health conditions, as well as health promotion and disease and injury prevention. Primary care is responsible for coordinating the care of patients and integrating their care with the rest of the health system by enabling access to other healthcare providers and services” [18].

### Purpose and objectives

The purpose of this paper is to describe models of care for the delivery of navigation services in primary care that has links to CBHSS. *‘Model of care’* is a multi-dimensional concept describing how healthcare services are organized and delivered [29]. Effective models create the conditions for people to receive the right care, at the right time, by the right team, and in the right place. We provide an overview of issues addressed, the subpopulations of clients with whom navigators work, role titles, and hiring and training of navigators. We also report specifically on characteristics of models as described in the literature, including location, type of navigator, patient populations, purpose and outcomes.

## Methods

### Search strategy

The purpose of our review is to understand the role of navigators and models of navigation in primary care settings with an emphasis on navigation that addresses inequities and social determinants of health. We chose to conduct a scoping review which is used when a synthesis of existing knowledge is needed to map key concepts and gaps in a defined field. As per this approach, we did not evaluate the methodological quality of studies as the focus was not to look solely at outcomes [30]. The overall search strategy followed the established methods for a scoping literature review [31, 32].Our strategy included four search activities including: i) an electronic database search; ii) a web site search; iii) key informant contacts and; iv) hand search of substantive literature reviews on the topic were conducted. Health sciences librarians worked with the research team to develop an electronic search strategy utilizing search terms from relevant articles to identify key words [1, 22, 27, 28, 33]. Relevant databases, including CINAHL, MEDLINE, EMBASE, PsychInfo, and CCTR were searched for literature published between 1990 to June 2013 using MeSH headings and free text key words that were applicable to two areas of interest: navigation and primary care. Terms were combined with Boolean operators ‘AND’ and ‘OR’. Next a general internet search using Google and Google Scholar was conducted.We also contacted one author and retrieved two additional articles [12, 34]. Examination of bibliographies of nine review articles resulted in the selection of potentially relevant papers.

Inclusion and Exclusion Criteria and the Review Process.

Papers retrieved from the electronic database search were subject to four levels of review/screening using inclusion/exclusion criteria (Table 1). To limit the yield of papers, we restricted the countries of origin of the study to Australia, Canada, New Zealand, the US, United Kingdom, and Western Europe. We used EndNote and Distiller SR to file and manage retrieved electronic papers and record reviewer decisions of the reviewers.

**Table 1:** Inclusion/Exclusion criteria for scoping review papers

Inclusion Criteria Published in English Published between 1990 to June 2013 Countries of origin of study: Canada, United States, United Kingdom, New Zealand, and/or Western Europe (may have involved multiple countries, but must include at least one of those listed) Must include the following: • Navigator or navigation process • Navigation role by professional or paraprofessional • Primary care setting • Navigation that involves the community (beyond the health care system) Was a published or unpublished primary study, descriptive paper, report, literature review using any type of method
Exclusion Criteria Published in language other than English Published before 1990 Countries of origin of study other than Canada, United States, United Kingdom, New Zealand, and/or Western Europe If navigation was a secondary outcome Article did not describe in detail the extent of community navigation Article did not address navigator or navigation process Article did not include a navigation role by professional or paraprofessional Article did not take place in a primary care setting Article is an editorial, commentary or book review

Prior to each stage of review of relevancy, team members collaborated for consensus building and consulted with librarians. The title and abstract of papers retrieved from the library database search were independently evaluated by two members of the research team (*n* = 5) in the first level of review. During this time, in order to avoid excluding potentially relevant papers, the research team included papers where there was insufficient information or doubt about relevancy. Any papers assessed as relevant by at least one member of the team progressed to the next level of review.

The second level of screening consisted of a full text review of each paper by two members of the team. To ensure papers were relevant to primary care, the reviewers used criteria in our chosen definition of primary care, and looked for a description of “a service at the entrance to the healthcare system. It addresses diagnosis, ongoing treatment and the management of health conditions, as well as health promotion and disease and injury prevention” [18]. In the case of disagreement at this stage, the researcher leads (RKV and NC) reviewed papers and reached consensus regarding inclusion. The team then elected to apply an additional level of screening for more detailed descriptions of navigation due to the large number of articles. Thus, the third level of screening consisted of a second full text review of included papers for specific descriptions of community navigation beyond the health care system, including community based health care and/or social services. For all relevant papers in the fourth level, members of the research team extracted data using both Distiller SR and a common data extraction form created by the team.

### Analysis

Data from all extracted papers were coded using NVivo 10 software and themes were identified. The coding structure was developed by the research team and was based on the research questions. Coding categories were refined and collapsed numerous times at research meetings. Specific information related to the characteristics of navigation delivery models were extracted and informed by Joshi and colleagues’ examination of the structure of primary care delivery models [35].

## Results

### Search strategy results

**Fig. 1:**
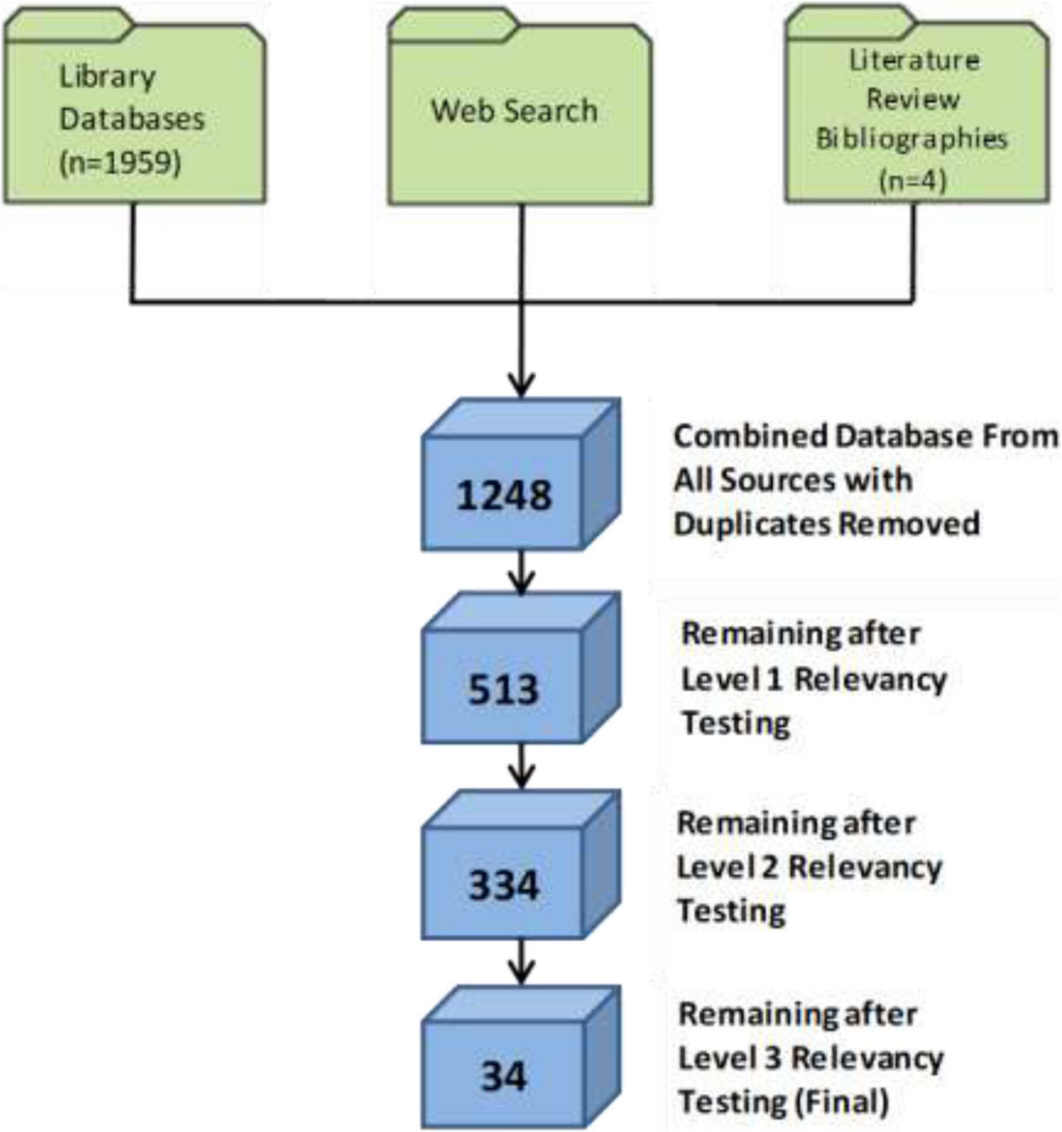
Search strategy and yield

Results of the overall yield of papers are presented in Fig. 1. Following this, we provide the countries of origin and methods used.

### Numbers, sources, and types of papers

Of the 34 papers included in the review, as shown in Table 2, most (85%) originated in the US. This is an expected finding given the emergence of navigation in the US. Table 2 also shows the range of research methods reported which are mostly non empirical descriptive papers (32.3%), followed by qualitative (26.4%) and quantitative research papers (20.5%). Of the quantitative papers, three were randomized controlled trials and one was a non-randomized controlled trial. Three papers had unstated methods.

**Table 2:** Yield of papers by country of origin and stated research method (*n* = 34)

		References
Country of Origin	US (*n* = 29)	[8, 10–16, 27, 33, 36–38, 40–46, 50–56, 58, 59]
	Canada (*n* = 2)	[48, 49];
	United Kingdom (*n* = 2)	[47, 57],
	Australia (*n* = 1)	[39]
Research Method	Descriptive (*n* = 11; 32.3%)	[10, 15, 33, 36, 38, 40, 41, 48, 53, 54, 59]
	Qualitative (*n* = 9; 26.4%)	[11, 13, 16, 39, 42, 45, 47, 50, 55] • *n* = 6 qualitative descriptive studies [11, 16, 42, 45, 50, 55] • *n* = 3 grounded theory studies [13, 39, 47]
	Quantitative (*n* = 7; 20.5%)	[8, 37, 43, 46, 51, 56, 57] • *n* = 3 randomized controlled studies [37, 43, 46] • *n* = 1 non-randomized controlled study [8] • *n* = 2 cross sectional studies [51, 56] • *n* = 1 program evaluation [57]
	Other (*n* = 4: 11.7%)	• *n* = 1 literature review [44] • *n* = 1 feasibility study [14] • *n* = 1 pilot study [27] • *n* = 1 retrospective longitudinal chart analysis [52]
	Unstated Methods (*n* = 3; 8.8%)	[12, 49, 58]

In what follows, we present data on three main areas; the key health and/or social issues addressed by navigators or navigation programs in the literature; the patient populations receiving navigation services, and; the role titles assigned to the program personnel. Then we present the models or frameworks of systems navigation and a summary of different types of these models. Finally, we present information on the process of hiring and training system navigators.

### Issues addressed by navigators

*Health Systems Issues* were addressed by navigators in 13 papers. Navigators assist with fragmentation of health and social health care system through various methods, including communication [10–12, 14], access to care [10, 13, 14], navigating the system and services [13, 15, 16], health insurance [12, 33, 36], inappropriate care delivery [33, 37, 38], clients without permanent providers [10], and the need for better transitions [39]. In eight papers, *Disease Specific Issues* were addressed. Examples include mental illness [11, 14, 16, 37, 40], substance abuse [14, 40], cancer [14, 33], chronic disease [41] and comorbidities [12]. Navigators address issues related to *the social determinants of health* and these were identified in eight papers including housing concerns [14, 36, 42], food insecurity [8, 14, 40], legal issues [36, 40], employment issues [36, 37], financial difficulties [12, 40], racism [40], and lack of social support [12]. *Patient Related Issues* included lack of basic needs [40], patient fears and beliefs [12], self-management [11], adherence [36], and appointment compliance [11].

### Client populations of focus

Five major categories of patient populations were described, but many programs defined their populations by more than one of these categories, including: a) individuals with specific diseases; b) ethno-cultural groups; c) individuals experiencing economic barriers; d) age groups; e) vulnerable populations, and f) underserved populations. *Individuals with specific diseases* included those with chronic diseases [8, 11, 16, 27, 41, 43–47], mental illness and addictions [27, 37, 39, 43, 48, 49], cancer [12, 36, 50, 51], diabetes [38, 42, 47], HIV-AIDs [15, 52, 53], pediatric asthma [38], and obesity [38]. *Ethno-cultural groups* included Hispanics [16, 38, 42, 51], racial-ethnic minorities [33, 54], African-Americans [55], refugees and immigrants [38], and rural Americans [56]. *Individuals experiencing economic barriers* included those identified as low income [11, 16, 33, 37, 40, 43, 53, 54] and uninsured patients [11, 13, 43, 54]. Populations were also described according to *age group* including older adults [44–46, 56, 57], parents or guardians of young children [58], children [38, 58], and adolescents [58].*Vulnerable populations* included homeless populations [10, 53], victims of intimate partner violence [38, 40], patients experiencing food insecurity [38], at-risk mothers [38], and unemployed veterans [53]. Three papers also described patient populations as *underserved* [13, 33, 54].

Unlike most other papers, Ferrante and colleagues provided detailed descriptors of patients requiring navigation services including clients who were: seeing multiple specialists; using internal resources frequently (high staff demands, frequent phone calls or visits); requiring social services; needing a difficult or complex referral, homebound, having family communication issues, or requiring mental health or pain management support [27].

### Role titles for navigators

In our review, we identified 15 different titles or terms for individuals providing navigation support, including Community Health Worker [11–13, 38, 42, 58], Community Health Liaison [55] or Community Health Advisor [13], Patient Navigator [13, 27, 33, 38, 50] or Navigator [41, 48], Case Manager [14, 39, 43, 52, 53], Promotoras [11, 41], Guided Care Nurse [8, 44–46], Healthy Families Brooklyn Advocate [13], Lay Health Advocate [13], Healthy Living Coach [38], Visiting Mom [38], Program Coordinator [38] and Specialist Nurse [59].

### Navigation models and frameworks

In 33 papers, a navigation model or framework was described. Table 3 lists the studies, organized by the program design (lay person, health professional, student or team navigation) and provides information reported about the location, purpose, model components, and reported or perceived outcomes of the navigation program. Reported or perceived outcomes have been categorized as patient outcomes (PO), provider or navigator outcomes (PNO) and health system outcomes (HSO) (Table 3).

**Table 3:** Characteristics of system navigation models and their purpose

Study	Location of study	Components of the model	Purpose	Reported or perceived outcomes^a^
Studies of lay person navigators (*n* = 12)				
Retkin et al., 2013	New York City, US	Name: Healthcare Legal Partnership (HeLP)	To improve health and well-being of vulnerable communities by integrating legal assistance in patient navigation	PO - improvements in general health and wellness, patients who were connected to legal services reported positive impacts on finances and even improved compliance with medical appointments and treatment PNO - increased understanding of the law and skills to address patient’s needs
		Type of Navigator: Lay patient navigator trained in legal issues with support of attorneys		
		Population: People with Cancer and HIV		
Esperat et al., 2012	Texas, US	Name: Transformacion Para Salud (Transformation for Health)	A chronic disease self-management model to develop a culturally sensitive intervention to facilitate patient behavior changes	PO - improvements in general health and wellness, Improved self-efficacy, self-management or empowerment HSO - Reduction in emergency room and/or hospital use
		Type of Navigator: Certified community health workers		
		Population: Underserved populations with chronic diseases		
Layne et al., 2012	Atlanta, US	Name: Good Samaritan Health Centre	To assist new patients in establishing a healthcare home, to prevent disease, detect health conditions at an earlier stage and provide more successful treatment, and reduce preventable ED visits	PO- Increased access to care HSO - Reduction in emergency room and/or hospital use
		Type of Navigator: Patient Navigator assisting with financial and medical system practices working with primary care providers		
		Populations: Uninsured adult patients living in poverty with no regular primary care provider		
Spiro et al., 2012	Massachusetts, US	Name: The MGH Chelsea community health improvement team	To provides support for everyone involved in patient care: patients, providers, the community at large, and the internal CHW staff	PO - improvements in general health and wellness, increased patient satisfaction PNO - satisfaction with navigation programs, opportunity to redevelop as professionals
		Type of Navigator: Community Health Worker		
		Population: Vulnerable sub-populations including refugees/immigrants, Latinos, an those facing significant economic, education and health challenges		
Brown et al., 2011	Brooklyn, US	Name: Healthy Programs Brooklyn	To increase access to care, improve health education and ease navigating the health care system	PNO - increased knowledge and skills
		Type of Navigator: Trained lay navigators		
		Population: Residents living in New York City housing authority developments		
Linkins et al. 2011	Minnesota, US	Name: Stay Well, Stay Working (SWSW)	To offer working persons with serious mental illness a comprehensive set of health, behavioral health, and employment support services	PO - improvements in general health and wellness, increased access to care, increased employment and reduced financial stresses, reduced numbers of mental health patients who applied for disability benefits, and a significantly higher percentage of behavioural health claims compared to controls
		Type of Navigator: Navigators trained in vocational rehabilitation serving an employment support role		
		Population: Social Security Disability beneficiaries with psychiatric illnesses		
Carroll et al., 2010	New York, US	Name: Cancer Patient Navigation Program	To assess and alleviate barriers to adequate health care	PO - Negative experiences were reported in a cancer patient navigation program delivered by community health workers (CHW), from a variety of settings including primary care
		Type of Navigator: Community Health Workers		
		Population: Newly diagnosed patients with breast or colorectal cancer		
Gimpel et al., 2010	Dallas, US	Name: Project Access Dallas	To provide access to health and social care for the “working poor” who are ineligible for existing, publicly-funded health care	PO - improvements in general health and wellness, Improved self-efficacy, self-management or empowerment, working poor served in one study noted that services were now affordable
		Type of Navigator: Community Health Workers (CHWs)		
		Population: Uninsured, low income residents requiring access to health care		
Clark et al., 2009	Boston, US	Name: Boston REACH 2010 Breast and Cervical Cancer Coalition Women’s Health Demonstration Project	To identify and reduce medical and social obstacles to breast cancer screening and following up abnormal results	PO - Increased access to care
		Type of Navigator: Case managers		
		Population: Women of African descent		
Mayhew et al., 2009	London, UK	Name Integrated Care Co-ordination Service (ICCS)	To provide supports to older adults to prevent hospital admissions and early admissions to long-term care	HSO - Reduction in emergency room and/or hospital use
		Type of Navigator: Care coordinators		
		Population: Adults age 65 and over with one or more chronic conditions		
McCloskey et al., 2009	New Mexico, US	Name: LA VIDA (lifestyle and values impacting diabetes awareness)	To reduce barriers to health and social services and supports for Hispanics living with diabetes	PO - improvements in general health and wellness, Improved self-efficacy, self-management or empowerment, Increased access to care PNO - empowered in their community advocacy role and some were promoted into supervisory roles
		Type of Navigator: *Promotores du salud* (community members who act as healthcare navigators) in a community health centres		
		Population: Hispanics with diabetes or at risk for diabetes		
Bradford et al., 2007	Portland, Seattle, Boston, Washington, US	Name: HIV Systems Navigation	To increase engagement and retention in HIV primary medical care for individuals previously unconnected or tenuously connected to care	PO - improvements in general health and wellness, Increased access to care
		Type of Navigator: Non-clinical staff with Bachelor’s degree in social science or healthcare		
		Population: HIV-infected individuals with co-occurring mental and substance abuse disorders		
Studies of Nurse Navigators (*n* = 10)				
Wolff et al. 2009; Boult et al. 2010; Foret Giddens et al. 2009; Boyd et al. 2007	Three mid-Atlantic health regions, US	Name: Guided Care Model	To improve the quality of life, quality of care, and efficiency of resource use for medically complex older adults To support caregivers of older adults with complex health-related needs; to improve patients’ health and the well-being of their families and friends	PO- Improved self-efficacy, self-management or empowerment, increased access to care, improvements in caregiver depression and strain PNO - satisfaction with navigation programs, increased trust, increased communication between primary care providers and community services, and among providers
		Type of Navigator: Guided Care Nurses (registered nurses) and interdisciplinary primary care team		
		Population: Medically complex older adults and caregivers of older adults		
Maeng et al. 2013	Rural central Pennsylvania, US	Name: Proven Health Navigator (PHN)	To provide chronic care and patient-centred primary care services in rural communities	PO- Increased access to care HSO- Reduction in emergency room and/or hospital use
		Type of Navigator: Nurse case managers		
		Population: Adults with severe or multiple chronic conditions requiring case management		
Kramer et al. 2012	Large Mid-western city, US	Name: Safe Mom, Safe Baby (SMSB)	To provide interdisciplinary case management to support pregnant women experiencing Intimate Partner Violence (IPV)	PO - improvements in general health and wellness
		Type of Navigator: Registered nurse and domestic violence advocate		
		Population: Marginalized women who self-disclose Intimate Partner Violence (IPV) who are pregnant or recently pregnant		
Burton et al., 2010	US	Name: Patient-centered chronic care management	To educate as a support to patients and their families, and facilitate access to community resources	PO- Improved self-efficacy, self-management and empowerment
		Type of Navigator: Nurse case managers		
		Population: Patients with primary immunodeficiency disease (PIDD)		
Williams et al., 2010	UK	Name: Community Matrons	To improve patient self-management and education, and enhance co-ordination between primary and social care.	PO - improvements in general health and wellness, access, patient advocacy, and psychosocial support.
		Type of Navigator: Advanced Practice Nurse		
		Population: people living with long-term conditions in the community		
McCann & Clark, 2005	UK	Name: Community mental health nurses	To promote wellness and caring	Outcomes not measured
		Type of Navigator: Community mental health nurses (registered nurses)		
		Population: Young adults with early episode of schizophrenia		
Pfeffer et al., 1995	San Diego, US	Name: Special Infectious Disease (SPID) Case Management Model	A framework to provide cost-effective, accessible, continuous, quality health care.	PO - Increased access to care
		Type of Navigator: Advanced Practice (Nurse practitioners)		
		Population: Veterans with HIV-related illnesses and AIDS		
Studies of Social Work Navigators (*n* = 1)				
Ferrante et al., 2010	US	Name: Patient Navigator pilot	To help patients use the health care system efficiently in primary care practices	PO - Increased access to care
		Type of Navigator: Social worker		
		Population: Elderly patients (mostly female)		
Studies of Student Navigators (*n* = 1)				
Bishop et al. 2009	Charlottesville, US	Name: Charlottesville Health Access (CHA)	To provide access to health and social services and connect homeless adults to permanent primary care services	None reported
		Type of Navigator: Medical and nursing students trained in navigation		
		Population: Homeless adults		
Studies of Navigation Delivered by Teams of Health Professionals and Lay Persons (*n* = 6)				
Tejeda et al. 2013	Chicago, US	Name: Chicago Patient Navigation Research Program (PNRP)	To identify and remove barriers faced by African-American and Latina women receiving cancer diagnoses and treatment	PO - Increased access to care
		Type of Navigator: Lay navigators and clinical social workers		
		Population: African-American and Latina women with breast or cervical cancer diagnoses without prior treatment		
Mullins et al., 2012	Baltimore, US	Name: Community Partnership Program	To foster community collaboration and raise awareness of the need to improve health in the community and to identify and connect patients to existing resources and services	PO increased patient satisfaction, increased access to care PNO - satisfaction with navigation programs, increased communication between primary care providers and community services
		Type of Navigator: Health care professionals, community health workers, faith-based ministries and community leaders		
		Population: African American and Hispanic communities		
Bohman et al., 2011	Houston, US	Name: The Texas Demonstration to Maintain Independence and Employment	To coordinate a set of health benefits and employment supports to help low-income, working adults maintain their employment and remain independent of publicly funded disability assistance	PO- improvements in general health and wellness, increased patient satisfaction, increased access to care, no differences in employment, hours worked or earnings
		Type of Navigator: Nurses, social worker and vocational specialists		
		Population: Uninsured working adults with chronic mental, behavioral and physical health conditions		
Hendren et al. 2011	Rochester, US	Name: Patient Navigation Research Program (PNRP)	To understand health disparities related to barriers to care for newly-diagnosed cancer patients	None reported
		Type of Navigator: Community health workers (CHW) and hospital and primary care teams		
		Population: Newly diagnosed breast and colorectal cancer patients		
Palinkis et al., 2011	California, US	Name: Multi-faceted Depression and Diabetes Program (MDDP)	To prevent depression relapse through chronic illness management interventions including problem solving treatment and patient/family education	None reported
		Type of Navigator: Patient navigator, social worker, psychiatric consultant		
		Population: Hispanic diabetic patients with depression		
Tataw et al. 2011	Los Angeles, US	Name: South Central Los Angeles Health Care Alliance (SCHCA)	To provide case management support aimed at empowering families to navigate the health care system	PO- Improved self-efficacy, self-management or empowerment, increased patient satisfaction, increased access to care
		Type of Navigator: Community health workers (CHW) and pediatric primary care teams		
		Population: Low-income urban children and families without a regular source of healthcare		
Studies of Navigation Delivered by Teams of Health Professionals (*n* = 3)				
Anderson et al. 2009; Anderson et al. 2009	Western Canada	Name: Sooke Navigator Project	To provide a community-based intervention to support access to mental health and social support services	PNO - increased communication between primary care providers and community services, increased trust
		Type of Navigator: Two navigators with training in social work and psychiatric rehabilitation		
		Population: Adults with mental health and addictions		
Halkitis et al. 2010	New York City, US	Name: AIDS Service Organizations (ASO)	To raise the level of health, mental health, and quality of life for HIV-positive women	None reported
		Type of Navigator: Case manager and interdisciplinary team of physicians, nurses, mental health professionals, social workers, and community representatives		
		Population: Black and Latina HIV-positive women		

^a^*PO* Patient outcomes, *PNO* Provider or navigator outcomes, *HSO* Health system outcomes

### Lay person navigator models

Twelve papers reported navigation models that involved a lay person as the navigator. This is defined as a non-professional who is trained to perform specific activities related to system navigation functions. The aim of most models was to provide general support to facilitate access to health care through linking and connecting [11, 13, 15, 54, 57] or removal of obstacles or barriers [14, 42, 50]. Some lay person delivered models were developed to address needs of specific populations such as women of African descent [14] or immigrants [38, 42]. For instance, promotoras were community members who acted as healthcare navigators to reduce health disparities among Hispanics in a diabetes intervention program in New Mexico [42].

In four models, the navigator was described as a community health worker (CHW) [38, 50], or a certified CHW [11, 41] which suggests a more formalized training program and role. CHWs worked with populations with chronic diseases [41] and newly diagnosed cancer [50]. Other models described navigators with specific skill sets including legal assistance expertise [36], financial practices [54], and vocational rehabilitation [37].

Patient outcomes reported in lay person led navigation studies included improved general wellness [11, 15, 37, 41, 42], reduced financial stresses [36, 37], increased employment [37] and improved knowledge and skills [36]. Other outcomes reported were reduction in emergency room or hospital use [41, 54, 57].

### Nurse navigator models

Ten papers described six navigation models that were led by nurses. Two of the models utilized Advanced Practice Nurses [47, 53] and four models utilized Registered Nurses [8, 39, 40, 44–46, 56, 59]. Four papers [8, 44–46] describe the Guided Care Model, a nurse-led interdisciplinary model of primary care designed to improve the quality of life and resource use for medically complex older patients. A detailed description of the role including training and competencies has been published [45].

All nurse led navigation models were developed for patient populations with complex needs such as patients with multiple chronic conditions [8, 44–47, 56], immunodeficiency diseases [53, 59] or young adults with schizophrenia [39]. The Safe Mom, Safe Babies program is a strengths-based nurse-led program providing services in healthcare settings for women who are victims of domestic violence [40]. Three studies reported increased access to care [44, 53, 56] and others report improved patient [45, 59] and caregiver outcomes [46].

### Team based navigation models

#### Teams of health professionals and lay persons

Six papers described navigation provided by teams comprised of health professionals and laypersons [12, 16, 40, 43, 51, 55, 58]. The issues being addressed by navigation teams were complex. For instance, Palinkas and colleagues [16] reported on a team made up of a patient navigator, social worker and psychiatric consultant working with Hispanic diabetic patients with depression. The model described by Bohman and colleagues [43] included a team of nurses, social workers and vocational specialists working with uninsured working adults with chronic mental, behavioural and physical health conditions. Patient outcomes reported in these six papers included increased access to care [40, 43, 55, 58], improvements in health and wellness, increased patient satisfaction [43, 55, 58] and improved self-efficacy, self-management or empowerment [58].

#### Teams of health professionals

In three papers, navigation services were provided by teams of health professionals. The Sooke Navigator Project in Western Canada was developed by a community-based steering committee. The goal of the project was to improve access to mental health and addiction services and increase communication between community-based providers, primary care and the mental health system [48, 49]. The model included one navigator with a background in social work and one with a background in psychiatric rehabilitation. Outcomes of the project included referral facilitation, increased access to assessments and more collaborative planning. Halkitis and colleagues [52] used a retrospective, longitudinal analysis of charts of HIV-positive women to understand the impact of case management and supportive services provided by interdisciplinary team of physicians, nurses, mental health professionals, social workers, and other community agency representatives on access to health care and fulfillment of basic needs (food, clothing, etc.) impacting health and quality of life. Results showed transportation, the use of primary health care and medical specialists, and a support group were the most frequently used services.

### Criteria and competencies of navigators at time of hire

Criteria and competencies that employers looked for when hiring navigators was described in 16 papers. *Experience requirements* of individuals included previous work with the patient population [8, 15, 45, 48, 49], community experience [8, 13, 53], and counselling [49]. *Skills requirements* included: skills in social work [48], coordination [55], health education [55], and computers [13]. Pfeffer and Schnack described the need for skills in problem-solving, conflict management and negotiation [53]. *Desirable personal traits* of navigators were reported in eight papers [8, 11, 13, 40–42, 48, 49]. These included strong effective communication skills, cultural competence, respect, enthusiasm for coaching, compassion, acceptance, reliability, dedication, flexibility, commitment to education, client-centeredness, ethical work and the ability to work with males or females and within groups. *Knowledge requirements* were reported in seven papers [13, 15, 36, 40, 48, 49, 55] and included: knowledge of the health care system, specific diseases and related community resources, mental health and addictions, legal issues, support services and bilingualism. Only three papers reported *education requirements* and these ranged from in house training to university degree [15, 41, 58].

### Training for system navigators

#### Content of training programs

Authors described the content of training and orientation programs for navigators in 22 papers. The most commonly reported content of training programs included information related to specific diseases [13, 14, 41, 42, 50, 51, 58], professional skills such as communication and customer service [12, 13, 42, 50, 51], case management training [14, 50, 58], information related to local community services and the utilization of health care services and resources [12, 42, 50, 51].

Three papers reported a specific educational program developed for nurses who provide care in the Guided Care Model [8, 45, 46]. The content included coaching, coordination of health care, group facilitation skills, making referrals and proactive primary care. Other training content related to populations at risk [10, 40], knowledge of the legal system [13, 36], and vocational rehabilitation [37, 43]. A number of papers reported providing theoretical content such as the Transformation for Health framework [41] and concepts of strengths-based practice [15], motivational interviewing [15], community health empowerment, public health and the “Rule of Three” (prevention, detection, management) [13], and proactive primary care [45]. One paper noted training to identify and remove barriers to care [51]. The Healthy Families Brooklyn Advocate, a lay health worker program included practical training on conducting and leading health fairs, as well as cardio-pulmonary resuscitation and First Aid training [13].

#### Training methods

Some papers described the delivery method and length of training programs. First we report on training for health professionals followed by training for lay navigators. Web-based delivery strategies were used to train professionals. Nurses in the Guided Care Model received a 40 h online course earning a Certificate in Guided Care Nursing from the American Nurse Credentialing Centre [44]. This course was developed by a multidisciplinary faculty team [45]. Promotoras, who are community workers, received 160 h of basic certification training and 6 weeks of chronic disease management training [41]. Video clips of intimate partner violence survivors, as well as simulated practice scenarios and web-based educational programming was utilized for healthcare provider navigators working with these victims [40].

Three papers reported on training for lay navigators or volunteers which showed that both active and passive learning strategies were used and included both in-person and online approaches. In a paper by Carroll and colleagues [50] lay navigators participated in an eight-week intensive training program, an 80 h family development course credentialed by New York State and a two to three day event sponsored by the American Cancer Society. Lay health workers in the Health Families Brooklyn Program received 30 h of didactic classroom training [13]. Lay navigators in the Chicago model learned through interactive role play, lectures and in-person and webinar training sessions [51].Volunteers in the Charlottesville Health Access program were nursing or medical students participated in a 90 min orientation seminar and received a Navigator Resource manual with up-to-date information about community services [10]. A number of papers reported on the need for ongoing learning and support for navigators [13, 38, 40, 45].

## Conclusion

Fragmentation of the health care system is an antecedent for the creation of navigator roles and navigation service delivery models in primary care. The purpose of this review was to scope out the current literature on navigation models and to provide a better understanding through a description of the roles of navigators and models of navigation within primary care that includes links to CBHSS. We found that various models of health delivery were employed for different populations. In particular, navigations models led by health care professionals and interprofessional teams were focused on addressing patient populations with complex health and social needs. Navigation models led by lay-persons were tailored to more stable populations with a central focus on social determinants of health. The multitude of diverse navigation models speaks to the complexity of client needs for health care and social service support in different populations and contexts. Roles and models have been developed to meet specific needs of populations ranging from the provision of primary care in nurse-led models [56] to the coordination of health benefits and employment support by lay persons [43].

Despite the concern that navigation roles can add complexity to the health care system [20, 60], the development of navigation roles and models speaks to unmet needs for coordination and facilitation of care and service, particularly in relation to populations for whom social determinants of health create additional barriers to accessing social and health care services and supports. We make an important contribution to the literature by highlighting navigation models that address both health and social service navigation. The emergence and development of system navigation signals an important shift in the recognition that health care and social care are inextricably linked especially to address the social determinants of health.

Given the high degree of variance in the literature included in this review, it is difficult to draw conclusions regarding the effectiveness of navigation or navigators in primary care (nor was it the purpose of the study), because only a small proportion of navigation interventions have been evaluated with suitable research designs. Only two models, the Guided Care Model [8, 44–46] and the Sooke Navigator project [48, 49] have been described more than once in the literature. For this reason, this scoping review was an instrumental approach to help researchers, policy makers, and clinicians traverse the breadth of current scholarship on navigation roles and models. This is an important first step in situating the use of system navigation in primary care, and is relevant to current reforms in primary health care to improve access to and coordination of health care for all populations [61]. We offer a review of the current state of knowledge of navigation roles and models, acknowledging the methodological limitations of the empirical research reviewed in this paper. Efforts to improve the rigour and comprehensiveness of navigation literature will help researchers to evaluate and synthesize outcome measures to determine effectiveness of navigation interventions, roles, and models.

Future studies of system navigation should examine the effectiveness of different types of models, roles or combination of roles in teams led by professional compared to lay navigators in facilitating improved services delivery for various patient populations and contexts and explore outcomes of navigation in primary care settings. Given the variety of primary care delivery and funding models globally, further research is needed to understand how different nations design and utilize system navigation programs within their primary care contexts. Due to the lack of maturation of the literature of navigation roles in primary care, the large number of descriptive studies included in this review can inform further innovation and development of navigation interventions and the development of performance and outcome indicators. The information presented regarding the issues addressed by navigators, the client populations, roles/titles, and the models of navigation can provide a foundation to inform the implementation of navigation roles in primary care. Another manuscript from this scoping literature review provides an overview of the implementation and maintenance of navigation roles and models [26].

## Abbreviations

CBHSS: Community-based health and social services
CHW: Community health worker
HSO: Health system outcomes
PNO: Provider or navigator outcomes
PO: Patient outcomes
